# Modelling of the Innate and Adaptive Immune Response to SARS Viral Infection, Cytokine Storm and Vaccination

**DOI:** 10.3390/vaccines11010127

**Published:** 2023-01-04

**Authors:** Cristina Leon, Alexey Tokarev, Anass Bouchnita, Vitaly Volpert

**Affiliations:** 1 Interdisciplinary Center for Mathematical Modelling in Biomedicine, S.M. Nikol’skii Mathematical Institute, Peoples Friendship University of Russia (RUDN University), 6 Miklukho-Maklaya St., 117198 Moscow, Russia; 2 M&S Decisions, 5 Naryshkinskaya Alley, 125167 Moscow, Russia; 3 Department of Foreign Languages No. 2, Plekhanov Russian University of Economics, 36 Stremyanny Lane, 115093 Moscow, Russia; 4 Semenov Institute of Chemical Physics, 4 Kosygin St., 119991 Moscow, Russia; 5 Bukhara Engineering Technological Institute, 15 Murtazoyeva Street, Bukhara 200100, Uzbekistan; 6 Department of Mathematical Sciences, The University of Texas at El Paso, El Paso, TX 79902, USA; 7 Institut Camille Jordan, UMR 5208 CNRS, University Lyon 1, 69622 Villeurbanne, France

**Keywords:** innate immune response, adaptive immune response, cytokine storm, vaccination, mathematical modeling

## Abstract

In this work, we develop mathematical models of the immune response to respiratory viral infection, taking into account some particular properties of the SARS-CoV infections, cytokine storm and vaccination. Each model consists of a system of ordinary differential equations that describe the interactions of the virus, epithelial cells, immune cells, cytokines, and antibodies. Conventional analysis of the existence and stability of stationary points is completed by numerical simulations in order to study the dynamics of solutions. The behavior of the solutions is characterized by large peaks of virus concentration specific to acute respiratory viral infections. At the first stage, we study the innate immune response based on the protective properties of interferon secreted by virus-infected cells. Viral infection down-regulates interferon production. This competition can lead to the bistability of the system with different regimes of infection progression with high or low intensity. After that, we introduce the adaptive immune response with antigen-specific T- and B-lymphocytes. The resulting model shows how the incubation period and the maximal viral load depend on the initial viral load and the parameters of the immune response. In particular, an increase in the initial viral load leads to a shorter incubation period and higher maximal viral load. The model shows that a deficient production of antibodies leads to an increase in the incubation period and even higher maximum viral loads. In order to study the emergence and dynamics of cytokine storm, we consider proinflammatory cytokines produced by cells of the innate immune response. Depending on the parameters of the model, the system can remain in the normal inflammatory state specific for viral infections or, due to positive feedback between inflammation and immune cells, pass to cytokine storm characterized by the excessive production of proinflammatory cytokines. Finally, we study the production of antibodies due to vaccination. We determine the dose–response dependence and the optimal interval of vaccine dose. Assumptions of the model and obtained results correspond to the experimental and clinical data.

## 1. Introduction

Despite medical and technological advances, infectious diseases represent the leading causes of mortality worldwide. According to the WHO data, respiratory infections appear in the list of primary causes of death globally [1], and its mortality rate has increased in the last decades [2,3]. The ongoing COVID-19 pandemic emphasizes the need for a deeper understanding of the interaction of respiratory infections and the immune response of the host, especially in the case of viral pathogens.

The immune response to viral infection has some generic properties common for all pathogens, but it also has some particular features corresponding to the specific type of virus [4,5]. In general, after the ingress of virus into the host, two forms of immune defense are triggered. The first one, innate immunity, is the immediate reaction mechanism to limit tissue damage and prevent viral spread. It is characterized by its broad specificity, which is mediated through certain cell types (e.g., macrophages), cytokines and chemokines [6].

Cells of the innate immune response recognize antigen-infected cells, exhibit cytotoxic activity and begin to rapidly produce interferon inhibiting virus replication [7]. The most important members of the superfamily that belong to type I are IFN-α/β, and only IFN-γ belongs to type II [8,9]. Both type I and II interferon have antiviral activity [10]. The functions of type III interferon largely coincide with the functions of type I interferon. Both of these groups modulate the immune response after the pathogen is detected in the body, their functions being mainly antiviral and antiproliferative. However, type III interferon is less inflammatory and exhibits slower kinetics than type I interferon. In addition, the immunomodulatory effect of type III interferon is limited [11,12].

Interferon affects the virus indirectly by triggering the transcription of a number of genes after binding to the corresponding receptor on the cell, which leads to the production of proteins that block the replication of the virus in this cell [13].

Non-specific innate immune response provide the organism with more time for the development of the adaptive immune response. Naive T- and B-lymphocytes, after their differentiating into more specific immune cells (e.g., CD8+ and plasma cells) contribute to the elimination of infected cells, to the secretion of pathogen-specific immunoglobulins and to the generation of immunological memory [14].

Respiratory disease viruses enter the host with inhaled liquid droplet or via direct contact with the nose or eyes with infected surfaces [15]. Once it reaches the respiratory tract, the virus begins to spread in the host by infecting epithelial cells. Antigen-presenting cells (APCs), such as dendritic cells (DCs), macrophages (MP) and monocytes are also subjected to the presence of infection [16]. Infected antigen-presenting and epithelial cells stimulate the secretion of various cytokines, including interferon, which modulates the functions of the immune system and induces antiviral defense [17]. It is worth mentioning that certain viruses, such as MERS-CoV, are capable of replicating in naive and activated human monocytes, macrophages and DCs [18]. This differentiates SARS-CoV and SARS-CoV-2 viruses, which abortively infect these cells [19,20].

Specific cells of the adaptive immune response T- and B-lymphocytes undergo clonal expansion stimulated in an antigen-specific manner. Naive T-lymphocytes from thymus and naive B-lymphocytes from the bone marrow [21] circulate throughout the lymphatic system until they meet the APCs. The latter carry MHC molecules on their surface through which they present antigen fragments (peptides). Some T-lymphocytes recognize the presence of foreign peptides due the affinity of their T-cell receptor (TCR) to the antigen [22]. This antigen presentation process stimulates the activation of T-lymphocytes, leading to their proliferation and differentiation, mainly in cytotoxic T-lymphocytes (CTL) or CD8+ cells, T helper cells or CD4+ cells, and regulatory T cells [23]. CD8+ cells, also known as T killers, are responsible for destroying infected cells, while T helper cells, when contacting naive B-lymphocytes, activate them and stimulate their proliferation [24]. Effector B-lymphocytes, also known as plasma cells, initiate the production of virus-specific immunoglobulins or antibodies [25].

Mathematical modelling is widely used for the investigation of the immune response to viral infections (see the literature review in [26]). The most recent immunological models are described in [27]. Antigen presentation by the MHC molecules is considered in [28] in the framework of a multi-scale model of immune response. An ODE system describing the innate immune response to the influenza virus was proposed in [29,30,31]. Other models, also proposed for the influenza virus, analyze the effects of the adaptive immune response, leaving aside the innate immune response and the role of APCs [32,33]. There are also more general models that describe the evolution of different infections considering the implicit effect of the host adaptive immune response [34]. In [35,36], the main stages of the immune response, including APCs, are considered. In [37], the authors propose a system of 15 differential equations with 48 parameters to predict the innate and adaptive immune response to the influenza A virus infection. It takes into account the role of APCs, including DCs and use delay differential equations (DDEs) to account for the time delays between viral infection, immune cell activation, and migration of immune effector cells between tissue and lymphoid compartments.

Recent articles have focused on the interaction between SARS-CoV-2 and the immune response. A model with three ODEs was proposed [38] to describe the elements of the adaptive immune response, such as CD8+, IgG and IgM. The DDE model in [39] contains 11 equations, including one for body temperature. The ODE models presented in [40,41] highlight the importance of natural killer cells in viral containment at the stage of innate immune response. In [42], the authors proposed a multi-scale model for the evaluation of various therapeutic strategies for the treatment of infected patients. A mathematical model that considers the immune response associated with COVID-19 reproduces the clinical observations recorded after carrying out the Cuban immunotherapy protocols [43]. However, these models, for the most part, based on the pre-existing models of influenza A virus, leave out some parts of the immune response, such as cytokines, including mainly interferon, or particular features of SARS-CoV-type viruses.

Proinflammatory citokines are intrinsically related to the immune response. During the COVID-19 pandemic, the acute increase in proinflammatory cytokine levels has become a common complication. Similarly, mathematical models of the immune response have been focused on the understanding the emergence of cytokine storm. In [44], the authors proposed a set of 15 ODEs, which includes an equation representing cytokines, in order to study this phenomenon. The ODEs system analyzed in [45] focuses on the balance between pro- and anti-inflammatory cytokines. A biochemical model was considered in [46] specifying cytokines involved in this process. In [47], the normal immune response to infection was simulated and, through the variation of the system parameters, the conditions in which cytokine storm can arise were established. Finally, a previous version of the cytokine storm model, evaluated in Section 3.3 of this study, was presented in [48]. Despite the importance of better understanding the cytokine storm, mathematical models in this area are still scarce.

In this work, we develop a model of immune response to SARS-CoV-2 infection beginning from the innate immune response. Further, we include the adaptive immune response, investigate cytokine storm and vaccination. Section 2 provides the proposed mathematical models and the parameters identification process is described. Section 3 includes the main results of this work. Section 3.1 is devoted to the study of the interaction between the viral infection and innate immune response. In Section 3.2, we analyze the dynamics of the innate and adaptive immune responses together and determine the influence of antibodies in the containment and elimination of the virus. In Section 3.3, we introduce proinflammatory cytokines in the model of the innate immune response in order to study the emergence of cytokine storm. Section 3.4 describes the pathway of antibody production due to vaccination. In Section 3.5, a sensitivity analysis of the parameters used in the models is carried out. We conclude with Section 4, where we discuss the biological interpretation of the results, the validation of the proposed models and their limitations.

## 2. Materials and Methods

Considering the complexity of the immune response to the antigen, we use in this study the multi-compartment modeling method. The kinetics of the immune response to viral infection is considered in consecutive mathematical models described below.

### 2.1. Mathematical Model of Innate Immune Response

**Figure A1:**
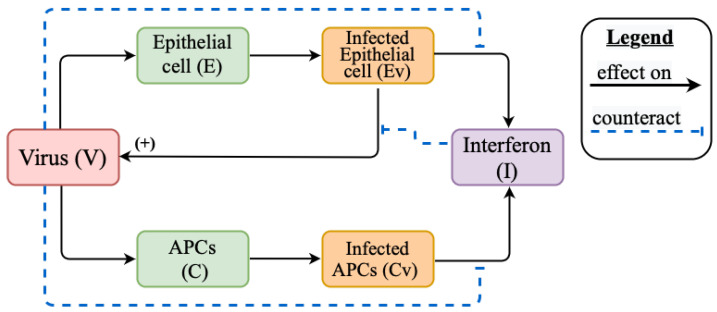
Schematic representation of the innate immune response to a viral respiratory infection. Green boxes indicate cells with constant presence in the organism. Orange boxes note virus-affected cells. Violet boxes show an induced immune response.

A pathogen entering the host initially confronts the first defense line of the organism constituted by the innate immune response. Adhering to *lex parsimoniae*, we schematize the innate immune response in Figure A1. This scheme contains the key aspects of the innate immune response, taking into account the peculiarities of SARS-type viruses, described in the introduction.

The following assumptions were considered for the innate immune response model. Infected epithelial cells produce new viral particles [49] and stimulate interferon secretion [17]. Viruses infect macrophages and dendritic cells [16]. SARS-CoV-infected macrophages and dendritic cells do not produce viral particles [19,20] but they stimulate interferon secretion [17]. The virus down-regulates interferon secretion [50], while interferon suppresses the production of viral particles by infected epithelial cells [50,51].

We consider the following system of differential equations for the interaction between the innate immune response and viral infection:

(1) $$\frac{dE}{dt}=k_{1}\left(E_{0}-E\right)-k_{2}EV,$$

(2) $$\frac{dE_{v}}{dt}=k_{2}EV-\sigma_{1}E_{v},$$

(3) $$\frac{dC}{dt}=k_{3}\left(C_{0}-C\right)-k_{4}CV,$$

(4) $$\frac{dC_{v}}{dt}=k_{4}CV-\sigma_{2}C_{v},$$

(5) $$\frac{dV}{dt}=f\left(I\right)E_{v}-\sigma_{3}V,$$

(6) $$\frac{dI}{dt}=g\left(V\right)\left(C_{v}+\kappa E_{v}\right)-\sigma_{4}I,$$

where

$$f\left(I\right)=\frac{f_{0}}{1+f_{1}I}\;,\;\;\;g\left(V\right)=g_{0}e^{-g_{1}V}\;.$$

In Equation (1) for the concentration of uninfected epithelial cells *E*, the first term in the right-hand side describes their influx proportional to the difference with the normal physiological value $E_{0}$. Epithelial cells are differentiated from basal cells which are not infected by the SARS-CoV-2 virus. Expression $k_{2}EV$ in this equation characterizes the rate of infection of uninfected cells by virus.

The same term determines the production rate of infected cells $E_{v}$ in Equation (2), while the second term in the right-hand side of this equation characterizes their death rate. Equations (3) and (4) for the concentrations of uninfected antigen presenting cells *C* and infected cells $C_{v}$ are similar to the previous two equations.

Equation (5) for the virus concentration *V* describes virus production by infected epithelial cells and virus death. The virus replication rate is inversely proportional to the interferon concentration *I*. Interferon down-regulates virus replication by activating the Jak-STAT signaling pathway [52]. The interferon kinetic is described by Equation (6) with its production by infected epithelial cells $\kappa E_{v}$ and mainly by infected APCs $C_{v}$, and its degradation $\sigma_{4}I$. Interferon production is down-regulated by the virus, as it is shown for SARS-CoV-2 [51]. Let us note that functions f(I) and g(V) are considered in different forms, the first one being inversely linear while the second one is exponential. This choice of down-regulating functions appears to be more appropriate for the analysis of this model. For the same starting decay rate at 0, the exponential function decays faster. This corresponds to more efficient interferon down-regulation by virus than the vice versa, which seems to be the case for SARS-CoV-2.

#### Innate Immune Response Model Reduction for the Study of Stationary Solutions

In this subsection, we carry out the model reduction to determine stationary points of system (1)–(6). Equating the right-hand sides of Equations (1)–(4) to 0, we obtain

$$E=\frac{k_{1}E_{0}}{k_{1}+k_{2}V}\;,\;\;E_{v}=\frac{k_{1}k_{2}E_{0}V}{\sigma_{1}\left(k_{1}+k_{2}V\right)}\;,\;\;C=\frac{k_{3}C_{0}}{k_{3}+k_{4}V}\;,\;\;C_{v}=\frac{k_{3}k_{4}C_{0}V}{\sigma_{2}\left(k_{3}+k_{4}V\right)}\;.$$

We substitute these expressions into Equations (5) and (6):

(7) $$\frac{dV}{dt}=f\left(I\right)\frac{k_{1}k_{2}E_{0}V}{\sigma_{1}\left(k_{1}+k_{2}V\right)}-\sigma_{3}V,$$

(8) $$\frac{dI}{dt}=g\left(V\right)\left(\frac{k_{3}k_{4}C_{0}V}{\sigma_{2}\left(k_{3}+k_{4}V\right)}+\kappa \frac{k_{1}k_{2}E_{0}V}{\sigma_{1}\left(k_{1}+k_{2}V\right)}\right)-\sigma_{4}I.$$

Set

$$f\left(I\right)=\frac{f_{0}}{1+f_{1}I}\;.$$

From Equation (7) for V≠0 we obtain

$$I=\frac{a_{1}}{1+b_{1}V}-d_{1}\equiv F\left(V\right)\;,$$

where

$$a_{1}=\frac{k_{2}E_{0}f_{0}}{f_{1}\sigma_{1}\sigma_{3}}\;,\;\;b_{1}=\frac{k_{2}}{k_{1}}\;,\;\;d_{1}=\frac{1}{f_{1}},$$

and from Equation (8)

$$I=Vg\left(V\right)\left(\frac{a_{2}}{1+b_{1}V}+\frac{a_{3}}{1+b_{2}V}\right)\equiv G\left(V\right)\;,$$

where

$$a_{2}=\frac{\kappa k_{2}E_{0}}{\sigma_{1}\sigma_{4}}\;,\;\;a_{3}=\frac{k_{4}C_{0}}{\sigma_{2}\sigma_{4}}\;,\;\;b_{2}=\frac{k_{4}}{k_{3}}\;.$$

Then from the equality F(V)=G(V), we get

(9) $$Vg\left(V\right)=\frac{\left(a_{1}-d_{1}\left(1+b_{1}V\right)\right)\left(1+b_{2}V\right)}{a_{2}\left(1+b_{2}V\right)+a_{3}\left(1+b_{1}V\right)}\;.$$

The study of stability of stationary points and dynamics of solutions is described in Section 3.1.

### 2.2. Mathematical Model of Innate and Adaptive Immune Response

**Figure A2:**
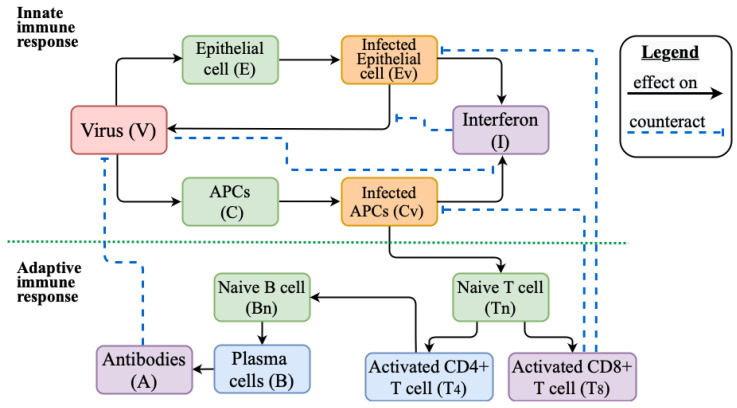
Schematic representation of the innate and adaptive immune responses to respiratory viral infection. Green boxes indicate cells with constant presence in the organism. Orange boxes note virus-affected cells. Blue boxes represent the cells responsible for stimulating the specific immune response. Violet boxes show effectors of innate and adaptive immune response.

After clonal expansion of T- and B-lymphocytes stimulated by APCs, the adaptive immune response acts to eliminate infected cells by means of cytotoxic T-lymphocytes (CTL) and neutralize free virions by antibodies. Figure A2 shows the joint action of innate and adaptive immune responses.

The following assumptions were considered for the model of adaptive immune response. The adaptive immune response begins with the CTL response from about day 6 after infection, and early antibodies appear from day 8 [53]. Infected antigen-presenting cells initiate an adaptive immune response by activating naive T-lymphocytes [54,55]. T-lymphocytes differentiate into CD4+ and CD8+ cells [23,56,57,58]. Activated cytotoxic T-lymphocytes eliminate cells infected by the virus [59,60]. Activated T helpers induce the differentiation of B-lymphocytes [24,61,62]. B-lymphocytes differentiate into plasma cells [63,64] which produce antibodies [25,65,66]. Antibodies recognize and neutralize foreign objects, such as viruses [67,68,69].

The interaction between the immune response and antigen (viruses of the SARS type) is modeled by the system of equations with two sub-systems. The first one corresponds to the innate immune response:

(10) $$\frac{dE}{dt}=k_{1}\left(E_{0}-E\right)-k_{2}EV,$$

(11) $$\frac{dE_{v}}{dt}=k_{2}EV-\sigma_{1}E_{v}-\gamma_{1}T_{8}E_{v},$$

(12) $$\frac{dC}{dt}=k_{3}\left(C_{0}-C\right)-k_{4}CV,$$

(13) $$\frac{dC_{v}}{dt}=k_{4}CV-\sigma_{2}C_{v}-\gamma_{2}T_{8}C_{v},$$

(14) $$\frac{dV}{dt}=f\left(I\right)E_{v}-\sigma_{3}V-\gamma_{3}AV,$$

(15) $$\frac{dI}{dt}=g\left(V\right)\left(C_{v}+\kappa E_{v}\right)-\sigma_{4}I,$$

and the second one to the adaptive immune response:

(16) $$\frac{dT_{n}}{dt}=h_{0}-h_{1}\left(C_{v}\right)T_{n}-h_{2}\left(C_{v}\right)T_{n},$$

(17) $$\frac{dT_{4}}{dt}=h_{1}\left(C_{v}\right)T_{n}-\sigma_{5}T_{4},$$

(18) $$\frac{dT_{8}}{dt}=h_{2}\left(C_{v}\right)T_{n}-\sigma_{6}T_{8},$$

(19) $$\frac{dB_{n}}{dt}=q_{0}-q_{1}\left(T_{4}\right)B_{n},$$

(20) $$\frac{dB}{dt}=q_{1}\left(T_{4}\right)B_{n}-\sigma_{7}B,$$

(21) $$\frac{dA}{dt}=k_{5}B-\sigma_{8}A-\gamma_{3}AV,$$

where

$$\begin{array}{c} f\left(I\right)=\frac{f_{0}}{1+f_{1}I}\;,\;g\left(V\right)=g_{0}e^{-g_{1}V}\;,\;h_{1}\left(C_{v}\right)=\frac{h_{1}^{0}C_{v}}{1+h_{1}^{1}C_{v}}\;,\\ h_{2}\left(C_{v}\right)=\frac{h_{2}^{0}C_{v}}{1+h_{2}^{1}C_{v}}\;,\;q_{1}\left(T_{4}\right)=\frac{q_{1}^{0}T_{4}}{1+q_{1}^{1}T_{4}}\;. \end{array}$$

Equations (10)–(15) correspond to the action of the innate immune response just as in the previous section, but now we take into account its interaction with the adaptive immune response. Thus, in Equation (11), the term $\gamma_{1}T_{8}E_{v}$ describes the removal of infected epithelial cells by CD8+ cells with an elimination rate $\gamma_{1}$. A similar meaning has the term $\gamma_{2}T_{8}C_{v}$ in Equation (13) with the parameter $\gamma_{2}$ that characterizes the elimination rate of infected APCs cells $C_{v}$ by CD8+ cells $T_{8}$. Equation (14) is completed by the term representing the elimination of free virions *V* by antibodies *A* with a rate constant $\gamma_{3}$.

Equations (16)–(21) characterize the dynamics of adaptive immunity. The pool of naive CD4 and CD8 cells we define as naive T-lymphocytes ($T_{n}$). In Equation (16), for the concentration $T_{n}$ of naive lymphocytes, $h_{0}$ characterizes their influx with a constant rate. They differentiate into CD4+ cells with concentration $T_{4}$ and CD8+ cells with $T_{8}$, with the rate depending on APC concentration $C_{v}$. The corresponding terms appear in Equations (17) and (18) followed by the terms describing cell death.

Equation (19) describes the concentration of naive B-lymphocytes $B_{n}$ with a constant production rate $q_{0}$. The second term describes the decrease in the concentration of these B cells due to their differentiation induced by T helper cells. We neglect cell death in Equations (16) and (19) since it is negligible in comparison with cell differentiation in the presence of antigen. In Equation (20), the term $q_{1}\left(T_{4}\right)B_{n}$ describes the production of plasma cells *B*, and the next term characterizes their death rate. Finally, Equation (21) corresponds to the kinetics of antibodies. Plasma cells *B* secrete antibodies with a production rate $k_{5}$. The antibody concentration in the host is reduced by its death rate $\sigma_{8}$ and their depletion in the process of virus neutralization. The analysis of this model allows us to evaluate the action of the innate immunity together with adaptive immunity to counteract respiratory virus infection of the SARS type.

### 2.3. Mathematical Model of Cytokine Storm

Cytokine storm or hypercytokinemia is a physiological reaction in which there is excessive stimulation of the innate immune system. Cytokines are mediators that participate in the inflammatory regulation of all branches of the immune system and can act locally as well as globally by intensifying their signal.

**Figure A3:**
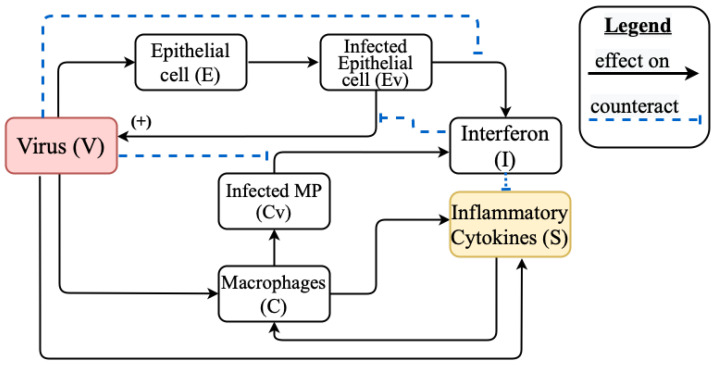
Representation of the mathematical model considered for the study of the emergency of the cytokine storm. The propose model is based on the model of the innate immune response by adding the corresponding member to describe the pro-inflammatory cytokines.

In the case of respiratory viruses, the infected epithelial cells and infected antigen presenting cells stimulate the secretion of interferon *I* as the first line of defense of the organism. Interferon in turn activates the secretion of pro-inflammatory cytokines *S*, such as IL-6, IL-1, IL-2, IL-7, IL-10, granulocyte colony-stimulating factor (G-CSF), IP-10, MCP1, macrophage inflammatory protein 1α (MIP1α) and tumor necrosis factor (TNF) [70,71]. The very presence of the virions also stimulates the secretion of these pro-inflammatory cytokines. These secreted pro-inflammatory cytokines are responsible for regulating the proliferation and influx of other cells of the immune system, such as dendritic cells, macrophages, prime adaptive T and B cells *C*, which in turn also contribute to the secretion of pro-inflammatory cytokines *S*. A dysfunctional immune response can cause this common modus operandi of innate immunity to incite a cascade reactions that can cause the hyper-production of cytokines [72]. This super strong reaction of the innate immune response can have a fatal outcome for the host, as it does not give way to adaptive immune response through cytotoxic T cells and T helpers and can induce programmed cell death (apoptosis, necroptosis, and autophaguia). Figure A3 visualizes the scheme of this interaction between cytokines and immune cells.

We take into account in the model that viral infection can stimulate production of inflammatory cytokines by infected cells and by the cells of the immune response [73,74]. Recent studies indicate that inflammatory cytokines can activate cell death, which in turn leads to further cytokine secretion [75,76].

We consider the following system of equations to characterize the action of inflammatory cytokines in innate immunity:

(22) $$\frac{dE}{dt}=k_{1}\left(E_{0}-E\right)-k_{2}EV,$$

(23) $$\frac{dE_{v}}{dt}=k_{2}EV-\sigma_{1}E_{v},$$

(24) $$\frac{dC}{dt}=\frac{r_{1}S}{1+r_{2}S}+k_{3}\left(C_{0}-C\right)-k_{4}CV,$$

(25) $$\frac{dC_{v}}{dt}=k_{4}CV-\sigma_{2}C_{v},$$

(26) $$\frac{dV}{dt}=f\left(I\right)E_{v}-\sigma_{3}V,$$

(27) $$\frac{dI}{dt}=g\left(V\right)\left(C_{v}+\kappa E_{v}\right)-\sigma_{4}I,$$

(28) $$\frac{dS}{dt}=\frac{r_{3}S}{1+r_{4}S}C+p\left(V,I\right)-\sigma_{S}S,$$

where

$$f\left(I\right)=\frac{f_{0}}{1+f_{1}I}\;,\;\;\;g\left(V\right)=g_{0}e^{-g_{1}V}\;,\;\;\;p\left(V,I\right)=\frac{p_{1}V}{1+p_{2}V+p_{3}I}\;.$$

The initial conditions for this system of equations and the values of parameters are given in Appendix A.2.

For the cytokine storm study, the main changes in the model of the innate immune response analyzed in Section 3.1 concern Equation (24) and an additional equation for pro-inflammatory cytokines (28). Pro-inflammatory cytokines stimulate the proliferation of macrophages with the rate $r_{1}S/\left(1+r_{2}S\right)$, as described in the first term in the right-hand side of Equation (24). In Equation (28), the first term characterizes the secretion of pro-inflammatory cytokines by macrophages stimulated by these cytokines. The second term corresponds to the secretion of pro-inflammatory cytokines stimulated by the presence of virus and down-regulated by interferon. Finally, the third term characterizes the clearance of pro-inflammatory cytokines. The analysis of this model allows us to determine conditions of normal and excessive inflammatory response, the latter interpreted as cytokine storm.

#### Model Reduction for the Study of Stationary Solutions

We begin to study system (22)–(28) with the analysis of stationary points. We obtain from the right-hand sides of these equations the following relations:

$$\begin{array}{cc} E=\frac{k_{1}E_{0}}{k_{1}+k_{2}V}\;,\;\; & E_{v}=\frac{k_{1}k_{2}E_{0}V}{\sigma_{1}\left(k_{1}+k_{2}V\right)}\;,\\ C=\frac{k_{3}C_{0}\left(1+r_{2}S\right)+r_{1}S}{\left(k_{3}+k_{4}V\right)\left(1+r_{2}S\right)}\;,\;\;\; & C_{v}=\frac{k_{4}V\left(k_{3}C_{0}\left(1+r_{2}S\right)+r_{1}S\right)}{\sigma_{2}\left(k_{3}+k_{4}V\right)\left(1+r_{2}S\right)}\;. \end{array}$$

Substituting them into Equations (26)–(28), we obtain

(29) $$\frac{dV}{dt}=f\left(I\right)\frac{k_{1}k_{2}E_{0}V}{\sigma_{1}\left(k_{1}+k_{2}V\right)}-\sigma_{3}V,$$

(30) $$\frac{dI}{dt}=Vg\left(V\right)\left(\frac{k_{4}\left(k_{3}C_{0}\left(1+r_{2}S\right)+r_{1}S\right)}{\sigma_{2}\left(k_{3}+k_{4}V\right)\left(1+r_{2}S\right)}+\kappa \frac{k_{1}k_{2}E_{0}}{\sigma_{1}\left(k_{1}+k_{2}V\right)}\right)-\sigma_{4}I,$$

(31) $$\frac{dS}{dt}=\frac{r_{3}S}{1+r_{4}S}\left(\frac{k_{3}C_{0}\left(1+r_{2}S\right)+r_{1}S}{\left(k_{3}+k_{4}V\right)\left(1+r_{2}S\right)}\right)+p\left(V,I\right)-\sigma_{S}S,$$

and from (31) we deduce

(32) $$\begin{array}{c} \sigma_{S}S=\frac{r_{3}S\left(k_{3}C_{0}\left(1+r_{2}S\right)+r_{1}S\right)}{\left(1+r_{4}S\right)\left(1+r_{2}S\right)\left(k_{3}+k_{4}V\right)}+p\left(V,I\right). \end{array}$$

The study of stability of stationary points and dynamics of solutions is described in Section 3.3.

### 2.4. Vaccination Model

The model of immune response developed in Section 2.2 can be used to study antibody production due to vaccination. Hence, we consider the immune response model without virus replication and without infected epithelial cells, which corresponds to the system of equations:

(33) $$\frac{dC}{dt}=k_{3}\left(C_{0}-C\right)-k_{4}CV_{ac},$$

(34) $$\frac{dC_{v}}{dt}=k_{4}CV_{ac}-\sigma_{2}C_{v}-\gamma_{2}T_{8}C_{v},$$

(35) $$\frac{dV_{ac}}{dt}=-\sigma_{3}V_{ac}-\gamma_{3}AV_{ac},$$

(36) $$\frac{dT_{n}}{dt}=h_{0}-h_{1}\left(C_{v}\right)T_{n}-h_{2}\left(C_{v}\right)T_{n},$$

(37) $$\frac{dT_{4}}{dt}=h_{1}\left(C_{v}\right)T_{n}-\sigma_{5}T_{4},$$

(38) $$\frac{dT_{8}}{dt}=h_{2}\left(C_{v}\right)T_{n}-\sigma_{6}T_{8},$$

(39) $$\frac{dB_{n}}{dt}=q_{0}-q_{1}\left(T_{4}\right)B_{n},$$

(40) $$\frac{dB}{dt}=q_{1}\left(T_{4}\right)B_{n}-\sigma_{7}B,$$

(41) $$\frac{dA}{dt}=k_{5}B-\sigma_{8}A-\gamma_{3}AV_{ac},$$

where

$$h_{1}\left(C_{v}\right)=\frac{h_{1}^{0}C_{v}}{1+h_{1}^{1}C_{v}},\;h_{2}\left(C_{v}\right)=\frac{h_{2}^{0}C_{v}}{1+h_{2}^{1}C_{v}},\;q_{1}\left(T_{4}\right)=\frac{q_{1}^{0}T_{4}}{1+q_{1}^{1}T_{4}}\;.$$

Here the main difference from the model proposed in Section 2.2 is the absence of the viral replication term in the equation of viral dynamics (35); the equations corresponding to the dynamics of epithelial cells are also absent since they are not infected and do not participate in virus replication. Furthermore, the equation for interferon concentration is also omitted since there is no virus replication where the interferon interferes.

### 2.5. Parameter Indentification

The model of innate immune response consists of 6 equations and 15 parameters. For cytokine storm modelling, 1 equation and 8 parameters are added to the innate immune response model. For a complete immune response model, we add 6 equations and 16 parameters to the model of the innate immune response, which correspond to the characteristics of adaptive immune response. Certain parameters correspond to the values established in [37], where a mathematical model of the immune response to influenza A virus is proposed. These parameters include initial number of epithelial cells ($E_{0}=5\cdot 10^{5}$ cells/mL), immune response cells ($C_{0}=10^{3}$ cells/mL), such as macrophages and dendritic cells, as well as the rate constant of virus neutralization by an antiviral antibody unit ($\gamma_{3}=0.004\;$(day)^−1^ (units/mL)). The death rate of uninfected and infected antigen-presenting cells ($k_{3}=0.001$ and $\sigma_{2}=2.9$ day^−1^, respectively). Other fixed parameters, such as death rates of infected epithelial cells ($\sigma_{1}=1.2$ day^−1^) and virus decay rate ($\sigma_{3}=1$ day^−1^) are established on the basis of Refs. [29,36].

Values of parameters of the adaptive immune response, such as the killing rate of infected epithelial cells by $T_{8}$ ($\gamma_{1}=1.19\cdot 10^{-3}$ (cells·day)^−1^) and T-helper cells’ differentiation rate ($h_{1}^{0}=1.51$ (cells·day)^−1^) are determined in the works of [77] and [78], respectively. The parameter corresponding to the rate of antibody death ($\sigma_{8}=0.04$ day^−1^) is determined in Refs. [35,37,79].

The value of the interferon degradation constant ($\sigma_{4}=1$ day^−1^) agrees with the half-life of type I interferon ≈16 h [80,81]. Established rates of T-helper cells elimination ($\sigma_{5}\;=\;0.023$ day^−1^) and cytotoxic T cells elimination ($\sigma_{6}=0.031$ day^−1^) are equivalent to ≈30.13 and ≈22.36 days, respectively. These values are within the confidence interval established for the lifetime of these cells [82]. The rate of effector B-cell elimination ($\sigma_{7}\;=\;0.028$ day^−1^) ≈24.75 days is consistent with that observed in [83].

In the model of cytokine storm, the rate constant of the elimination of proinflammatory cytokines ($\sigma_{S}=0.25$ day^−1^) is equivalent to a lifetime of 66 h. This approximation falls within the observed elimination interval of various cytokines [84].

The next group of parameters was established on the basis of the values given in other works. The value for the death rate of uninfected epithelial cells ($k_{1}=4\cdot 10^{-3}$ day^−1^) allows us to investigate the case of the bistability of the system. This value retains the order of a similar parameter in [37]. The virus production rate ($f_{0}=1900$ (cells · day)^−1^ (copies/mL)) is four times higher than a similar value in the work of [35]; however, this is consistent with the observation that about $10^{3}$–$10^{4}$ viral particles are released from one infected cell in a day. The value of interferon secretion rate by infected cells ($g_{0}=500$ (pg/mL) (cells · day)^−1^) is four times less than in the same work. We consider such assumptions to be permissible since the current values of the parameters do not differ radically from those previously estimated in similar works. In addition, given the peculiarities of SARS-CoV viruses and the construction of the model itself, these parameters may be different.

We note that for the primary qualitative assessment of the inflammatory response of the organism in a cytokine storm, due to the generality of the model, the values of some parameters are estimated for the first time. All parameter values are varied to assess their role on the behavior of the system. The influence of each of the parameters on results is evaluated and detailed below.

## 3. Results

### 3.1. Innate Immune Response

#### 3.1.1. Stationary Solutions and Dynamical Behaviour

The variation of parameters around the reference values allowed us to identify different regimes in the infection progression.

**Figure 1:**
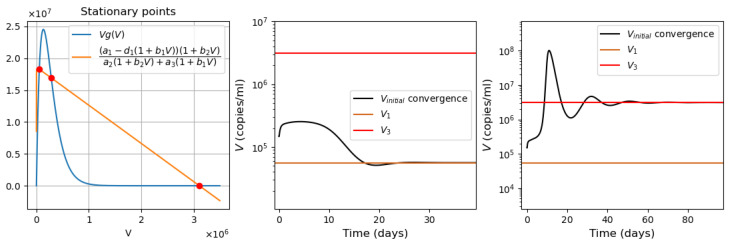
**Left**: graphical solutions of Equation (9) in the bistable case. **Middle**: convergence to the first stationary point, initial viral load $=1.48\cdot 10^{5}$. **Right**: convergence to the third stationary point, initial viral load $=1.5\cdot 10^{5}$.

**Figure 2:**
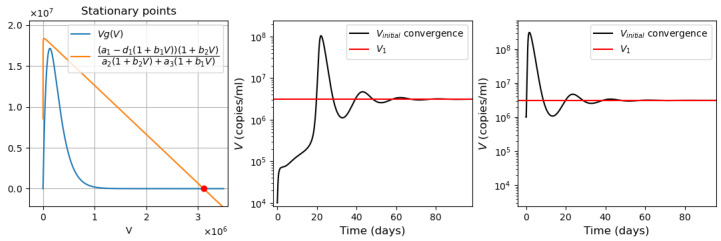
**Left**: graphical solution of Equation (9) in the monostable case with a large stationary value. Convergence to single large stationary point. **Middle**: initial viral load $=10^{4}$. **Right**: initial viral load = $10^{6}$. Interferon secretion rate $g_{0}=350$.

**Figure 3:**
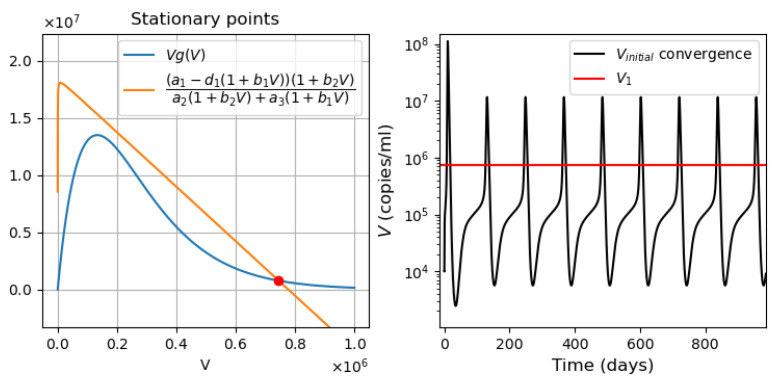
**Left**: graphical solution of Equation (9). **Right**: numerical simulations in the case of periodic oscillations with the initial viral load = $10^{4}$, interferon secretion rate $g_{0}=275$, influx rate of uninfected epithelial cells $k_{1}=0.001$.

- *Virus bi-stability.* Applying the estimated values from Appendix A Table A1 in Equation (9), we determine the presence of three positive stationary points. This corresponds to the case of system bistability, where the first and third stationary points are stable. The virus concentration is essentially larger in the second point compared with the first one. The system bistability implies different dynamics depending on the initial viral loads (Figure 1).
- *Virus monostability with a large stability value.* The case with a single stable point and large virus concentration is realized for a sufficiently small interferon production rate ($g_{0}\leq 373$, here and further, the dimensions of the parameters are indicated in Table A1, Table A2, Table A3 and Table A4 in Appendix A) or for a small virus clearance rate ($\sigma_{3}\leq 0.75$). Decreasing the value of $\sigma_{3}$ increases the stationary virus concentration. As might be expected, the increase in interferon clearance rate ($\sigma_{4}\geq 1.34$) or in turn the increase in the virus production rate ($f_{0}\geq 2.53$) also lead the system to this type of stability. Characteristic of this stability case is the appearance of a large virus peak with either a low or high initial viral load. For higher initial viral load, the peak is larger, and it is reached faster (Figure 2).
- *Virus monostability with a small stability value.* For a small virus production rate ($f_{0}\leq 1.54$ or $k_{1}\leq 0.0031$), the system becomes monostable with a small stability value. Low virus influence on interferon production ($g_{1}\leq 0.0048$) or high interferon influence on virus production ($f_{1}\leq 0.0008$) can also induce this effect.
- *Periodic oscillations.* If we decrease at the same time the values of $k_{1}$ and $g_{0}$, the system manifests periodic dynamics. As can be seen in Figure 3, the position of the stationary point coincides with that of Figure 2, which corresponds to monostability. However, because the value of the stationary point is not large enough, the kinetics of the system becomes characterized by a periodic behavior. The simulations in this case lead us to deduce that the period of oscillations decreases for smaller interferon production rate $g_{0}$.

Other parameters, such as the infection rate of epithelial cells by virus $k_{2}$, death rate of uninfected APCs $k_{3}$, infection rate of APCs by virus $k_{4}$ and death rate of infected APCs $\sigma_{2}$, are less essential in the sense that significant changes in their values cause only slight changes in the stationary values and do not affect their type of stability. The decrease in $\sigma_{1}$ causes a significant increase in the large stationary value. On the contrary, increasing the value of $f_{1}$ causes the small stationary value to decrease even more in the cases of monostability with a small stability value or the first stable point in bistability.

#### 3.1.2. Infection Dynamics with the Innate Immune Response

The system of Equations (1)–(6) was solved numerically with an ordinary differential equation solver (solve_ivp) using the Runge–Kutta method of the fourth-order accuracy with error estimates less than $10^{-8}$ in the programming language Python. The parameter values and initial values of variables are described in Appendix A, Table A1 and Table A4, respectively.

Convergence to the stationary solution takes approximately t=100 time units, though the convergence rate depends on the initial viral load. It should be noted that the virus monostability cases with small and with big stability values adopt the behaviors that correspond to the first and third stable points in bistability, respectively. Due to this, we will focus on the analysis of the behavior of the system in the bistable case. In addition, this case is more appropriate from the point of view of the dependence of the solution on the initial viral load.

**Figure 4:**
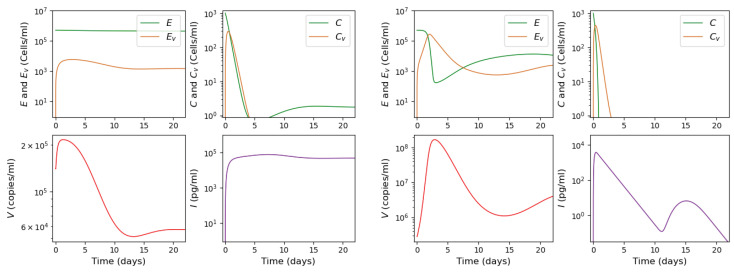
Numerical simulations of system (1)–(6). Behavior of solution for different initial viral loads in the bistable case. **Left** two columns: initial viral load $V\left(0\right)=141\cdot 10^{3}$ leads to the convergence to the first stable stationary solution. **Right** two columns: initial viral load $V\left(0\right)=28\cdot 10^{4}$ leads to convergence to the second stable stationary solution. The colors of the curves correspond to the colors of rectangles in Figure A1.

In the case of a low initial viral load (V(0) < 148,325), the system converges to the first stable point with the maximal viral load reached approximately after 1.5 days. The presence of infection stimulates the production of interferon that rapidly reaches a high concentration and stabilizes there. The moment of the maximal interferon concentration corresponds to the inflection point on the virus graph, after which the virus concentration tends to its stationary value. The lower the viral load is, the faster the virus converges to its stationary value with a low virus concentration in the organism. An example of such behavior is shown in the left panel of Figure 4.

**Figure 5:**
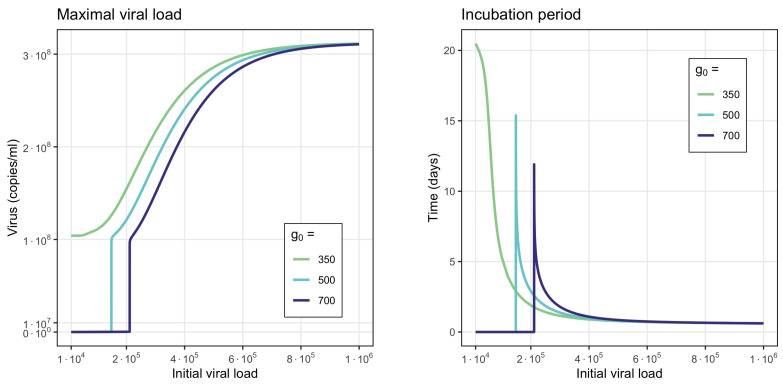
Maximal viral load (**left**) and incubation period (**right**) as functions of the initial viral load in numerical simulations of system (1)–(6). The vertical lines are the viral load values at which the system switched in convergence from the first to the third stationary point. The violet line corresponds to the monostable case with one large stationary point.

In the case of high initial viral loads (V(0)≥ 148,325), solution converges to the second stable stationary solution with behavior related to observed clinical manifestations. With small initial viral load, there is certain incubation period before the virus concentration begins noticeable growth. The higher the initial viral load is, the shorter the incubation period. It reaches 25.67 days for V(0) = 148,325. Additionally, a higher initial viral load leads to a larger peak in virus concentration. Figure 5 allows us to visualize these statements.

One of the important properties of the considered model is that the incubation period and maximal viral load depend on the initial viral load (Figure 5). For a high interferon production rate ($g_{0}=700$), the initial viral loads resulting in the change from the first to the third stationary point should be also sufficiently large. For small initial viral loads, the system remains at low virus concentrations without a peak of the viral load and without a clearly identifiable incubation period. For this value of $g_{0}$, transition occurs for $V\left(0\right)\approx 2\cdot 10^{5}$. A further increase in the initial viral load leads to the increase in the maximal viral load and to a decrease in the incubation period.

On the other hand, a low interferon production rate ($g_{0}=350$) leads to monostability with a high stable stationary value. Since any initial viral load converges to a single stable point, after reaching its peak concentration, there is no threshold value. A similar system behavior is observed for different values of the coefficient $f_{1}$, characterizing the interferon influence on the virus production rate.

Therefore, we can deduce that a high initial viral load not only shortens the incubation period but also stimulates a more intensive virus multiplication with a larger viral load. These conclusions are consistent with clinical observations of hospitalized and non-hospitalized patients carried out during the SARS-CoV-2 epidemic [85,86]. Regarding interferon, its secretion is rapidly stimulated by infection with a maximum of its concentration attained shortly after that. However, an increase in the initial virus load leads to a decrease in interferon production. We can see an example of such a behavior in Figure 4: for the initial viral load $141\cdot 10^{3}$, the interferon concentration reaches the value $7.62\cdot 10^{4}$, while with the initial viral load $28\cdot 10^{4}$, the maximum of interferon reaches $3.84\cdot 10^{3}$.

### 3.2. Innate and Adaptive Immune Response

The results of the previous section describe the reaction of the innate immune system to respiratory infections. However, the innate immune response operates alone until about days 6–8 post infection, with the adaptive immune response emerging after that [53].

The values of parameters of system (10)–(21) are specified in the Appendix A in Table A1 for the innate and Table A2 for the adaptive immune response. Some parameter values corresponding to the adaptive immune response are unknown and they are determined in numerical simulations based on the predicted behavior of the adaptive immune response.

**Figure 6:**
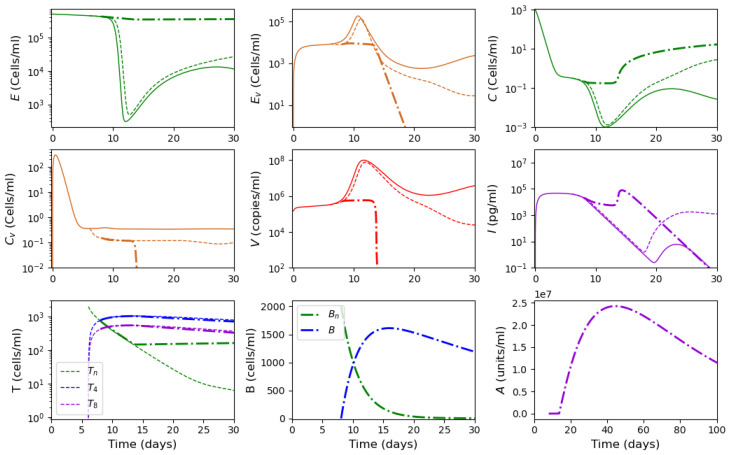
Numerical simulation of innate and adaptive immune response in system (10)–(21). The color code corresponds to Figure A2. Figures in the first two rows for the variables $E,E_{v},C,C_{v},V$ and *I* show the action of the innate immune response only (solid lines), innate immune response with CTLs beginning from day 6 (dashed lines) and with antibodies from day 8 (dash-dot lines). The figures of the last row show the T cells, B cells and antibodies. The initial conditions and parameter values are specified in Table A1, Table A2 and Table A3. Other values: initial viral load = $1.495\cdot 10^{5},\;k_{5}=1205.63$.

As described in [53], the adaptive immune response begins with cellular response approximately from day 6 post infection, while early antibodies are produced from day 8. Based on this information, Equations (16)–(18) are incorporated into the system from day 6 of the simulated time, and Equations (19)–(21) from day 8. Figure 6 shows an example of numerical simulations of the joint action of innate and adaptive immune responses.

As previously described in Section 3.1 for the innate immune response, the system behavior differs for low and high initial viral loads. In the case of a low viral load, the virus concentration remains sufficiently small, while the interferon concentration is high. In the case of a high initial viral load, the virus concentration attains high values after the incubation period, which depends on the initial viral load and other parameters.

**Figure 7:**
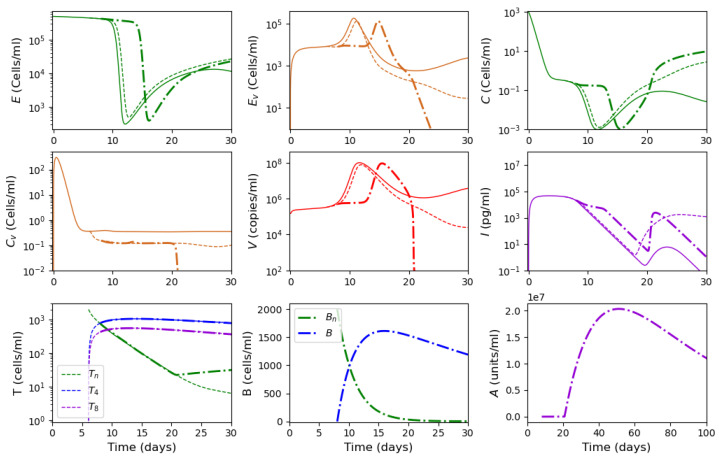
Numerical simulations of innate and adaptive immune responses in system (10)–(21). Notation is similar to Figure 6. A slight decrease in the parameter $k_{5}=1205.62$ essentially changes the dynamics of the system due to transitioning to another regime.

We study how the adaptive immune response influences the dynamics of viral infection. Analysis of solutions of system (10)–(21) shows that antibodies play a key role at this stage of immune response. It should be emphasized that the action of lymphocytes from day 6 along with the T killers and T helpers already causes a decrease in the maximum concentration of the viral load and slows down the development of the disease (Figure 6 and Figure 7, dashed lines). However, the mere action of T cells is not sufficient to contain the infection. The containment of the infection depends on the antibodies.

The antibodies efficacy in virus elimination can be regulated through the antibody production rate $k_{5}$. For the high initial viral loads, if the incubation period is greater than 6 days, it can be shown that a strong and effective adaptive immune response can reduce the virus concentration even before the peak of infection appears, as observed in Figure 6. On the other hand, if the adaptive immune response is not effective enough, i.e., the antibody production coefficient $k_{5}$ is not large enough, the incubation period can be extended up to three times. However, since the adaptive immune response fails to eliminate the virus, a high virus concentration is reached with a pronounced peak (maximal viral load) with the values about $10^{8}$ (copies/mL), in agreement with the clinical data (Figure 7).

Thus, Figure 6 and Figure 7 show two different dynamics of the adaptive immune response for the same innate immune response with a threshold transition determined by the value of $k_{5}$. For larger values of this parameter, the immune response successfully stops infection and does not allow it to progress to high virus loads. In the second case, for slightly smaller values of this parameter, the adaptive immune response increases the incubation period, but it stops infection progression only after a large virus outbreak.

**Figure 8:**
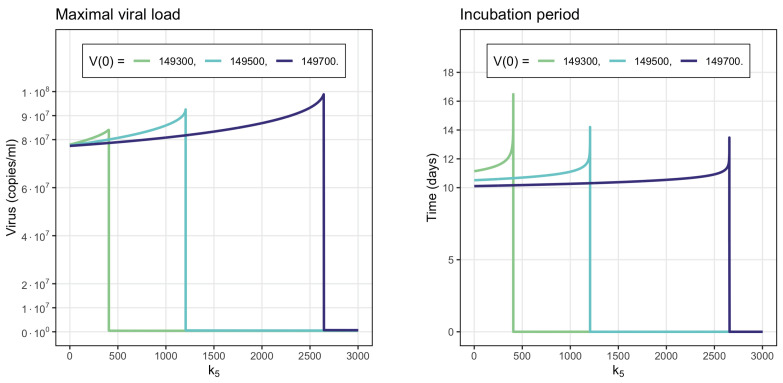
Numerical evaluations of the behavior of the virus depending on the antibodies secretion rate $k_{5}$. The vertical lines are the $k_{5}$ values at which the antibodies do not allow the development of viral infection, preventing the appearance of peaks with high virus concentrations. **Left**: maximal virus load. **Right**: incubation period.

Figure 8 shows the dependence of the maximal viral load and of the incubation period on the parameter $k_{5}$, characterizing the antibody production rate. We consider three different values of the initial viral load, for which the incubation period, as noted in Section 3.1, is slightly greater than 10 days. This selection of initial viral loads allows us to evaluate the influence of the parameter $k_{5}$ in the incubation period. For each of them, there is a critical value of parameter $k_{5}$ with very different behavior of the system below or above it (e.g., $k_{5}=1205.63$ for $V\left(0\right)=1.495\cdot 10^{5}$). If the antibody production rate is below the critical value, then the maximal viral load increases with the increase in this parameter. This seemingly paradoxical result is explained by the fact that antibodies cannot stop infection progression but increase the incubation period. The latter leads to the increase in the maximal viral load (Figure 7). If the antibody production rate exceeds the critical value, then they stop infection progression and the outbreak of the viral load is not observed (Figure 6).

Increase in the initial viral load decreases the incubation period and, as a consequence, decreases the maximal viral load. The critical value of the parameter $k_{5}$ increases with the increase in the initial viral load.

### 3.3. Cytokine Storm

#### 3.3.1. Stationary solutions

To simplify the analysis of Equation (32), to start, we consider the case p(V,I)=0.

**Figure 9:**
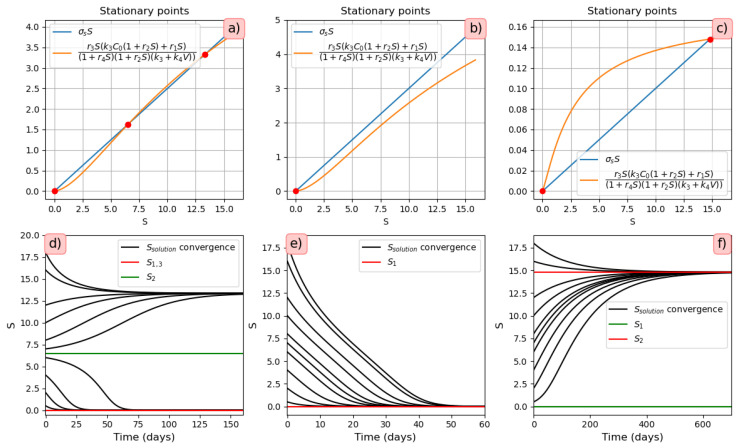
Graphical solutions of Equation (32) with V=0,p(V,I)=0 and the values of parameters (**a**) $r_{1}=3,\;r_{2}=0.1,\;r_{3}=1,\;r_{4}=0.1,\;\sigma_{S}=0.25$; (**b**) $r_{1}=3,\;r_{2}=0.1,\;r_{3}=1,\;r_{4}=0.1,\;\sigma_{S}=0.3$; (**c**) $r_{1}=1.4,\;r_{2}=0.8,\;r_{3}=1,\;r_{4}=0.5,\;\sigma_{S}=0.01$. Figures (**d**), (**e**) and (**f**) show the convergence to the stable stationary points (**a**), (**b**) and (**c**) respectively.

The parameter values correspond to the values used in the innate immune response model and are given in Appendix A, Table A1. Let *V* be a constant. Then, depending on the values of the parameters $r_{1},\;r_{2},\;r_{3},\;r_{4}$ and $\sigma_{S}$, we obtain three cases when solving Equation (32):

- One stationary point (Figure 9b). It is globally asymptotically stable. The solution converges to 0 under all initial conditions (Figure 9e).
- Two stationary points (Figure 9c). Stationary point S=0 becomes unstable, while the positive stationary point is stable. For any positive initial condition, the solution converges to the positive stationary point (Figure 9f).
- Three stationary points (Figure 9a). The convergence of the solution to the first or third stationary points depends on the initial value of S(0). If the initial condition is less than the value of the second stationary point (green line $S_{2}=6.53$), then the solution converges to the first stationary point $S_{1}=0$. For all other initial conditions, the solution converges to the third stationary point $S_{3}=13.35$ (Figure 9d).

The proposed parameter values allow us to observe cases of mono- and bistability. Taking into account the above parameters, we now proceed to study the behavior of the system at p(V,I)>0.

#### 3.3.2. Different Regimes of Inflammatory Response

**Figure 10:**
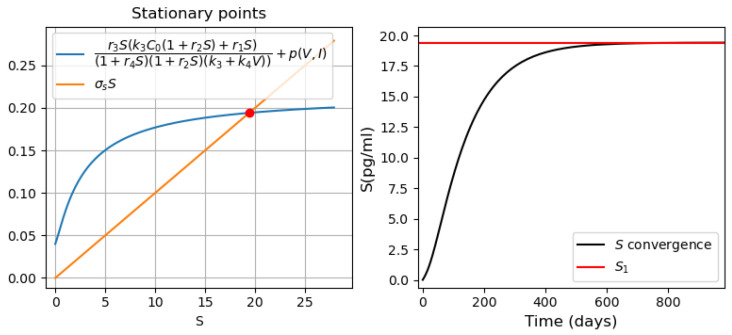
**Left**: graphical solution of Equation (32) in monostable case. **Right**: convergence of the concentration of proinflammatory cytokines to their stationary value, S(0)=0. The values of parameters: $r_{1}=1.4,\;r_{2}=0.8,\;r_{3}=1,\;r_{4}=0.5,\;\sigma_{S}=0.01,\;p_{1}=0.4,\;p_{2}=10,\;p_{3}=0.2$.

**Figure 11:**
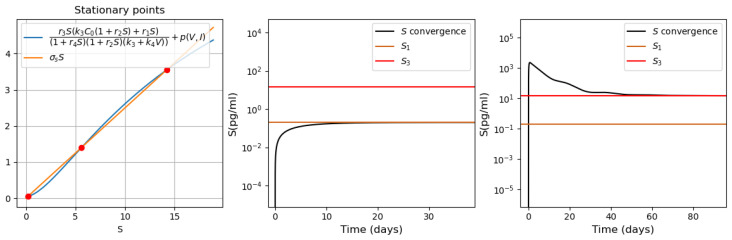
**Left**: graphical solution of Equation (32) in the bistable case. **Middle**: convergence of proinflammatory cytokines to the first stable stationary point, C(0)=0. **Right**: convergence of proinflammatory cytokines to the second stable stationary point, $C\left(0\right)=10^{3}$. The values of parameters: $r_{1}=3,\;r_{2}=0.1,\;r_{3}=1,\;r_{4}=0.1,\;\sigma_{S}=0.25,\;p_{1}=0.4,\;p_{2}=10,\;p_{3}=0.2$. Initial S=0.

In this section, we analyze the dynamics of the behavior of the system of Equations (22)–(28) to study the cytokine storm, assuming the initial concentration of the virus given ($V\left(0\right)=2.6\cdot 10^{5}$). We vary the concentration of immune response cells producing inflammatory cytokines. Depending on the value of the parameters, this system may have one or two stable stationary points (Figure 10 and Figure 11).

Let *V* and *I* be positive constants. Then from (32) and Figure 9, we can conclude that all stationary points take positive values other than zero.

- *Monostability of the system with pro-inflammatory cytokines.* For the parameter values used in Figure 10 (on the left), there is a single positive stationary point. The solution of Equation (28) corresponding to pro-inflammatory cytokines converges to this stationary value (Figure 10, right). The choice of the initial viral load affects only the time of convergence of the solution to a stationary value.
- *Bistability of the system with pro-inflammatory cytokines.* For the parameter values used in Figure 11, there are three positive stationary points. The initial condition C(0)=0 corresponds to the zero concentration of uninfected dendritic cells and macrophages. Under this initial condition, the concentration of S(t) converges to the first stationary value of $S_{1}=0.2$ (Figure 11, middle). When the initial concentration changes $C\left(0\right)=10^{3}$, the concentration of pro-inflammatory cytokines *S* converges to the third stationary value $S_{3}=14.208$ (Figure 11, right). It should be noted that $C\left(0\right)=10^{3}$ is the initial value used in the study of the innate immune response.

**Figure 12:**
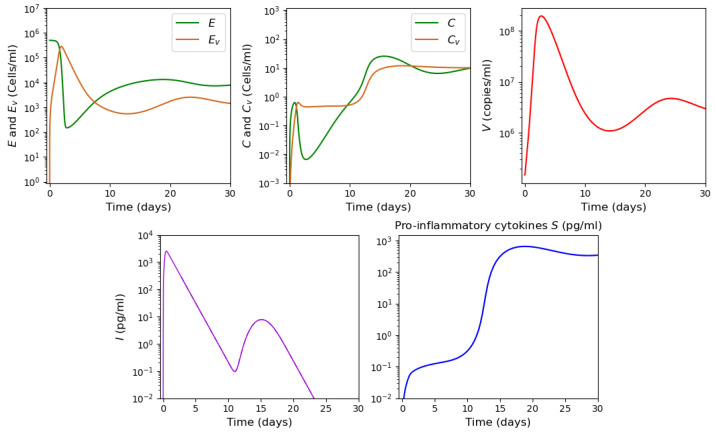
Numerical simulation for system (22)–(28). The values of parameters for the numerical experiment are detailed in Appendix A Table A1 and Table A3. The initial conditions correspond to those listed in Appendix A Table A4.

Note that the bistable case is observed for the value of the parameter $p_{1}\in \left(0;1.78\right]$. At the same time, if $p_{1}\in \left(0;1.59\right]$, then the concentration of *S* pro-inflammatory cytokines tends to the first stationary point. If $p_{1}\in \left[1.6;1.78\right]$, then the concentration of S(t) converges to the third stationary point regardless of the initial concentration of macrophages *C*. In this case, with the values of the parameters indicated in Table A1 and Table A3, there is a rapid increase in the concentration of pro-inflammatory cytokines to the maximum value corresponding to the third stationary point. If the infection rate of APCs decreases (e.g., $k_{4}=10^{-6}$), the concentration of S(t) first approaches the first stationary point with a low concentration of proinflammatory cytokines, and then moves to the third stationary point with a high concentration of pro-inflammatory cytokines. In Figure 12, the results of the numerical solution of the system (22)–(28) are presented for the parameter value $k_{4}=10^{-6}$. Thus, cytokine storm may not occur immediately, but at a certain stage of the development of the disease.

#### 3.3.3. Systemic Inflammation

Inflammatory cytokines can provoke inflammatory cell death that leads to the production of even more inflammatory cytokines [75]. This positive feedback loop can influence the onset and progression of the cytokine storm. In order to study this effect, we complete the previous model by the equation for the concentrations *R* of cells susceptible to inflammatory death:

(42) $$\frac{dR}{dt}=-a_{1}RS+k_{5}\left(R_{0}-R\right).$$

The first term in the right-hand side of this equation describes cell death due to the action of inflammatory cytokines, while the second term characterizes cell influx or proliferation till its concentration reaches its normal physiological value. The equation for the concentration *S* of inflammatory cytokines contains an additional term corresponding to the increase in their concentration due to cell death:

(43) $$\frac{dS}{dt}=\frac{r_{3}S}{1+r_{4}S}C+a_{2}RS+p\left(V,I\right)-\sigma_{S}S.$$

Let us note that the depletion of the cytokine concentration due to the interaction with cells is compensated by its larger production due to cell death. All other terms in this equation and all other equations remain the same as before. For simplicity, we consider here this interaction term in the form of the mass action law. All conclusions below remain valid in the case of saturation kinetics S/(1+kS) with respect to the concentration of inflammatory cytokines.

Consider, first, the case where the concentration of inflammatory macrophages *C* and the concentration of virus *V* equal 0. Then, Equation (43) becomes as follows:

(44) $$\frac{dS}{dt}=a_{2}RS-\sigma_{S}S.$$

Together with Equation (42), they form a closed system for the two concentrations. This system has two stationary points: $R=R_{0},S=0$ and $R_{1}=\sigma_{S}/a_{2},S_{1}=k_{5}\left(R_{0}-R_{1}\right)/\left(a_{1}R_{1}\right)$. Hence, there are two possible cases:

- If $\sigma_{S}>a_{2}R_{0}$, then $R_{1}>R_{0}$ and $S_{1}<0$. In this case, the point $\left(R_{0},0\right)$ is globally asymptotically stable. All solutions of system (42), (44) with non-negative initial conditions converge to it. The concentration of inflammatory cytokines, if is initially non-zero, exponentially decays.
- If $\sigma_{S}<a_{2}R_{0}$, then $R_{1}<R_{0}$ and $S_{1}>0$. In this case, the point $\left(R_{0},0\right)$ becomes unstable, while the point $\left(R_{1},S_{1}\right)$ becomes stable. Solution of system (42), (44) with any positive S(0) converges to it.

In the second case, inflammatory cell death is initiated by any small concentration of inflammatory cytokines. Hence, in normal physiological conditions, $\sigma_{S}>a_{2}R_{0}$, and inflammatory cell death does not occur. However, the values of these parameters remain the same during inflammation. Therefore, we conclude that $\sigma_{S}>a_{2}R_{0}$, even during viral infection.

We can now describe a different scenario of behavior of solutions of the complete model (22)–(27), (42), (43) with the corresponding biological interpretations. If system (22)–(28) (without inflammatory cell death) describes a cytokine storm, then the introduction of inflammatory cell death increases it due to additional cytokine production. If system (22)–(28) describes a normal inflammatory response, then additional cytokine production due to inflammatory cell death can move it to a cytokine storm.

On the other hand, if the virus is eliminated from the organism and proinflammatory macrophages vanish, then inflammatory cell death cannot sustain the cytokine storm by itself. The concentration of inflammatory cytokines will decay.

Thus, inflammatory cell death can reinforce the cytokine storm or initiate it from the normal inflammatory response, but it cannot initiate it or keep it going without an innate immune response.

### 3.4. Vaccination

In this version of the model, APCs described by the variable *C* are not infected but they still serve as antigen-presenting cells and stimulate the adaptive immune response and production of antibodies specific to the pathogen through plasma cells *B*.

**Figure 13:**
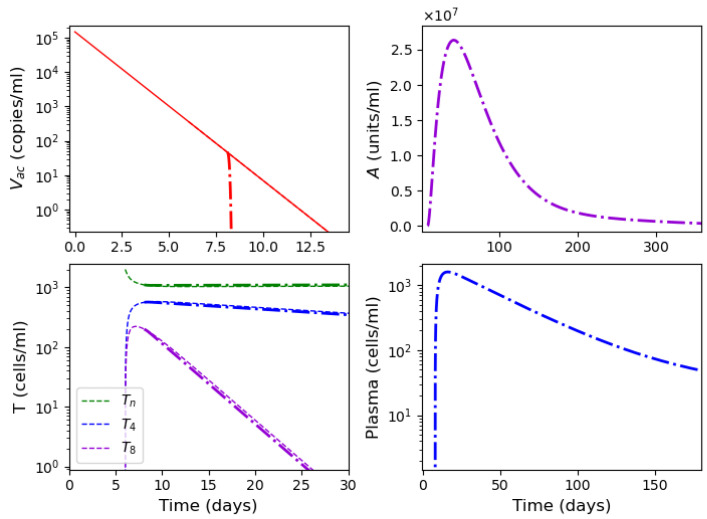
Numerical simulation of system (33)–(41) with virus concentration (**upper left**), antibodies (**upper right**), T cells (**lower left)** and plasma cells (**lower right**). Adaptive immune response (dash-dot lines) starts on day 6 and antibody production from day 8. Note the difference in time scales (along the x-axes) in all graphics.

Figure 13 shows the concentration of virus, antibodies and lymphocytes in numerical simulations of system (33)–(41). In the beginning of the simulations, the virus concentration decays exponentially since there is no virus replication. From day 8, it is neutralized by the antibodies.

The graph of antibody concentration is extended to 360 days for a better visualization of its dynamics. The maximal antibody concentration is reached after about 90 days and then their concentration gradually decreases. Similar dynamics are observed for plasma cells secreting antibodies.

**Figure 14:**
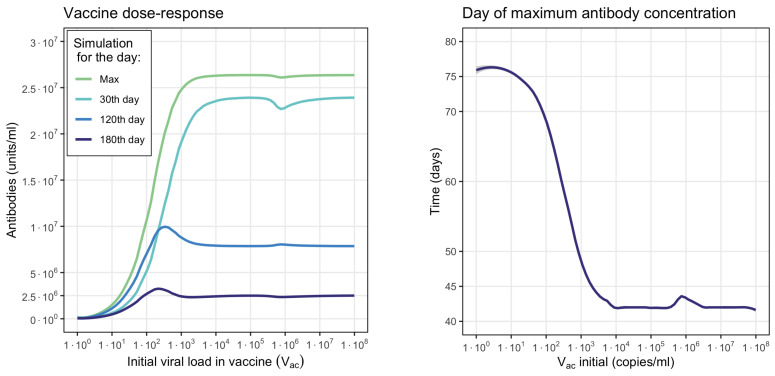
Numerical evaluations of antibody production depending on the vaccine dose ($V_{ac}$ initial). **Left**: maximal antibody concentration on 30th, 120th and 180th days after vaccination. **Right**: time to reach the maximal concentration of antibodies depending on vaccine dose.

Figure 14 shows the dose–response curve with the maximal antibody concentration depending on vaccine dose and antibody concentrations 1, 4 and 6 months after vaccination. According to the simulations, the optimal vaccine dose is in the range between $10^{4}$ and $10^{5}$ (copies/mL) where the antibodies reach maximum concentrations in a minimum period of time equivalent to approximately 42 days. A further increase in the vaccine dose can even decrease the antibody concentration, which is known as the Goldilocks effect [87,88].

### 3.5. Sensitivity Analysis

**Figure 15:**
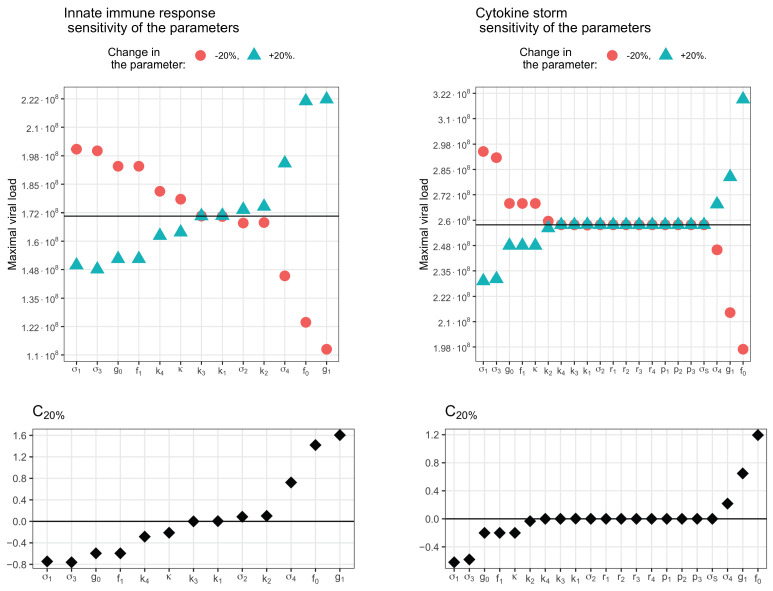
Sensitivity analysis of the innate immune response model (**left**) and the cytokine storm model (**right**) to the parameters. **Upper row**: the maximum viral load with an increase of 20% (triangles) and a decrease of 20% (dots) of each parameter compared to its value from the control set of parameters (solid line). **Lower row**: the corresponding sensitivity coefficients calculated by the formula $C_{20\%}=\frac{V_{+20\%}^{max}-V_{-20\%}^{max}}{0.4\cdot V^{max}}$. The control set of parameters is specified in the Table A1, Table A3 and Table A4. In all calculations, the initial value of the viral load was set to $V\left(0\right)=28\cdot 10^{4}$ (copies/mL). In the cytokine storm simulations, we used the value $k_{4}=10^{-6}$ day^−1^ (copies/mL).

Sensitivity analysis allows us to rank the parameters of the model according to the degree of influence on certain characteristics of its behavior. In this analysis, we changed each parameter individually by ±20%, in relation to the observed value that was the maximum concentration of the virus. By changing the latter, we calculated the sensitivity coefficients (Figure 15). When the parameters were changed to ±10%, the results were similar, with only minor changes (not shown).

With an innate immune response (Figure 15, left), increase of the rate of virus influence on interferon production ($g_{1}$) and of the virus production rate ($f_{0}$) significantly increases the peak concentration of the virus in the body, and the corresponding sensitivity coefficients are the largest. An increase in the interferon elimination rate ($\sigma_{4}$) has a slightly smaller effect. On the other hand, an increase in the values of parameters, such as death rate of infected epithelial cells ($\sigma_{1}$), virus decay rate ($\sigma_{3}$), interferon secretion rate ($g_{0}$) and rate of interferon influence in virus production ($f_{1}$), leads to a decrease in the maximum concentration of the virus. Less influence is exerted by infection rate of APCs by virus ($k_{4}$) and the relative activity of infected epithelial cells during interferon secretion describing by (κ). Parameters $k_{3}$, $k_{1}$, $\sigma_{2}$ and $k_{2}$ do not affect the maximum virus concentration.

In the cytokine storm model (Figure 15, right), all sensitivity coefficients are about a quarter less than in the innate immune response model. The most influential parameters remain the the virus production rate ($f_{0}$) and the rate of virus influence in interferon production ($g_{1}$), the interferon elimination rate ($\sigma_{4}$) has a lesser effect. $\sigma_{1}$, $\sigma_{3}$, $g_{0}$, $f_{1}$ and κ have an inhibitory effect. The infection rate of APCs by virus ($k_{4}$) and death rate of infected APCs ($\sigma_{2}$) cease to affect the maximum concentration of the virus in this model. The sensitivity to the infection rate of epithelial cells by virus ($k_{2}$) remains small but changes its sign to the opposite.

For a complete model of innate and adaptive immune responses, a control set of parameters is indicated in the Table A1, Table A2 and Table A4 with the exception of the antibody secretion rate parameter $k_{5}=1200$ [(cells·day)${}^{-1}$(units/mL)] and initial viral load $V\left(0\right)=1.495\cdot 10^{5}$ (copies/mL).

**Figure 16:**
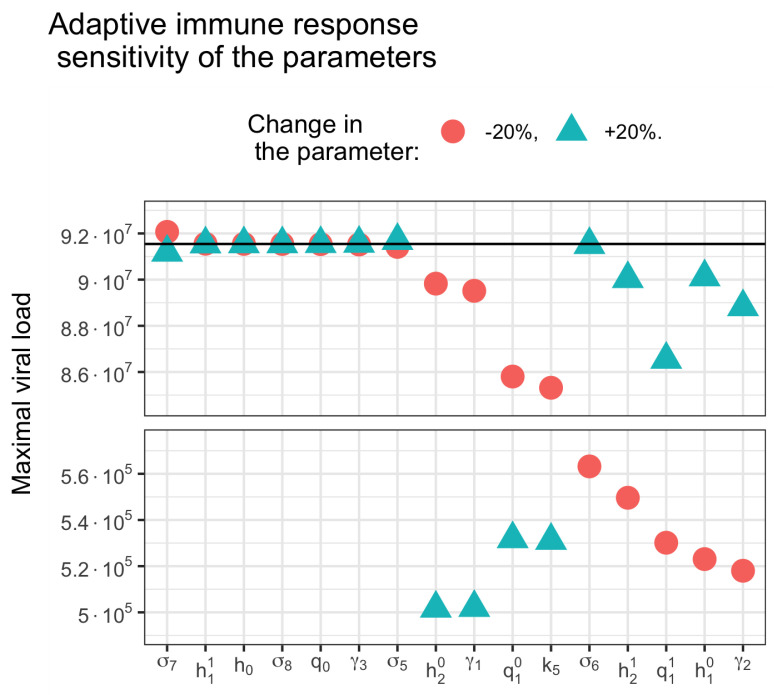
Sensitivity analysis of the adaptive immune response model parameters. Maximal viral load with an increase of 20% (triangles) and a decrease of 20% (dots) of each parameter compared to its value from the control set of parameters (solid line).

This set of values corresponds to the dynamics of adaptive immune response shown in Figure 7, in which an increase in the incubation period is observed; however, the development of the infection stops only after the peak of infection. In addition, this specific set of parameters is close to the boundary of the existence of the two observed regimes (with/without virus peak). For this reason, in Figure 16, it is observed how changing the parameter in one direction entails a sharp transition to another mode. If we change the parameter in the other direction, then the mode will remain the same, but this mode will have a decrease in viral load. This can be explained by the fact that as we move away from this regimen, the viral load decreases.

The parameters $h_{1}^{1}$, $h_{0}$, $\sigma_{8}$, $q_{0}$, $\gamma_{3}$ and $\sigma_{5}$ do not affect the maximum concentration of the virus, while $\sigma_{7}$ has little effect. Varying other parameters entails switching between the modes. An increase in the values of the parameters cytotoxic T cells (CD8+) differentiation rate ($h_{2}^{0}$), killing rate of infected epithelial cells by $T_{8}$ ($\gamma_{1}$), effector B-cells differentiation rate ($q_{1}^{0}$) and antibody secretion rate ($k_{5}$) prevents a viral peak (Figure 16, lower part).

A similar effect is observed with a decrease in the values of the parameters antibodies clearance rate ($\sigma_{8}$), cytotoxic T cells (CD8+) differentiation rate ($h_{2}^{1}$), effector B-cells differentiation rate ($q_{1}^{1}$), T-helper cells (CD4+) differentiation rate ($h_{1}^{1}$) and killing rate of infected APCs by $T_{8}$ ($\gamma_{2}$). At first glance, the observation regarding the $\gamma_{2}$ parameter may seem contradictory; however, it should be remembered that it is the infected APCs that trigger the acquired immune response by activating naive T cells.

## 4. Discussion

The main objective of this work was to study the interaction between the immune response and SARS-CoV type viruses. When studying the innate immune response, we observed different modes of infection development depending on the initial viral load and parameters of the immune response. We showed that in the case of a low initial viral load, the innate immune response stops the development of the infection even without an adaptive immune response. In this case, the infection is present in the body, but the viral load is not high enough and the disease is asymptomatic or with mild symptoms. In particular, we showed that the duration of the incubation period can vary in large limits. These observations agree with what was recorded in the meta-analysis carried out in [89] where the shortest mean incubation reported was 1.8 days and the longest incubation was 18.87 days. Furthermore, the initial viral load determines infection progression possibly leading to low or high values of maximal viral load [90]. This difference in the course of the disease depending on the initial viral load highlights the role of preventive measures (masks, social distancing, etc.), which, although they cannot completely eliminate the possibility of infection, can reduce the initial viral load and the severity of the disease [91].

At the second stage of this study, we included the adaptive immune response after 6–8 days post infection. We observe how the adaptive immune response can either stop infection progression, mainly due to virus elimination by antibodies, or if their action is insufficient, increase the incubation period and maximal viral load. This increase in the maximum viral load leads to an increase in the total viral concentration and therefore a more severe course of the disease.

One of the most relevant questions during the COVID-19 pandemic has been understanding the cytokine storm. Therefore, in Section 3.3, we extended the innate immune response model by introducing pro-inflammatory cytokines to study the occurrence of a cytokine storm. The inflammatory response to a viral infection can have different dynamics, with either low levels of inflammatory cytokines or high levels, or coexistence of both. The latter is the most interesting case with switching between two stable equilibria. As shown in Figure 12, inflammation stimulated by a viral infection can go to high levels, corresponding to a cytokine storm, and remain there even with a decrease in viral load. Also note that inflammation can slow down the progression of the infection due to an increase in the concentration of immune cells [92].

It should be noted that the physiological processes leading to the emergence of a cytokine storm are still not well understood. According to [73,93,94], the interaction of a viral infection with cells of the innate immune response can lead to their modification and a shift toward a pro-inflammatory phenotype that contributes to the development of a cytokine storm. In order to take into account the appearance of modified cells in the cytokine storm model, we assume in the calculations the initial concentration C=0 (Figure 12), bearing in mind that these macrophages differ from normal macrophages involved in the innate immune response. Note that in this model, we studied the emergence of a cytokine storm without taking into account the adaptive immune response. This simplification allowed us to reveal the main features of these process without unnecessary complications. The influence of the adaptive immune response on the cytokine storm will be considered in future studies.

Finally, the model proposed in Section 3.4 allowed us to study the production of antibodies due to vaccination. This model is built on the basis of the model studied in Section 3.2 omitting the terms and equations related to viral replication. Simulations show the dose–response curve and allow us to establish intervals of maximum immune response to different viral loads in the vaccine. We also observed that higher viral loads in the vaccine dose can lead to decreased antibody concentrations, which is qualitatively consistent with the search for the Goldilocks effect in vaccine doses [87,88].

The models developed in this work have certain limitations. We restrict the number of cytokines and immune cell types in order to avoid excessive complexity of the model. On the basis of systematic analysis presented in this work, we can introduce some other features of the immune response, such as regulatory T cells, cross immunity, etc., in the future investigations.

## Data Availability

Not applicable.

## Appendix A. Models Schemes, Parameters and Stationary Solutions Figures

### Appendix A.2. Model Variable Definitions and Initial Values

**Table A1:** Parameters for innate immune response.

Parameter	Value	Definition
$E_{0}$	$5\cdot 10^{5}$ [37]	Initial number of epithelial cells (cells/mL)
$C_{0}$	$10^{3}$ [37]	Initial number of dendritic cells
		and macrophages (cells/mL)
$k_{1}$	$4\cdot 10^{-3}$ [37]	Death rate of uninfected epithelial cells (day^−1^)
$k_{2}$	$7\cdot 10^{-8}$	Infection rate of epithelial cells
		by virus [day^−1^ (copies/mL)^−1^]
$k_{3}$	0.001 [37]	Death rate of uninfected APCs (day^−1^)
$k_{4}$	$10^{-5}$	Infection rate of APCs
		by virus [day^−1^ (copies/mL)^−1^]
$\sigma_{1}$	1.2 [29,37]	Death rate of infected epithelial cells (day^−1^)
$\sigma_{2}$	2.9 [37]	Death rate of infected APCs (day^−1^)
$\sigma_{3}$	1 [27,37]	Virus decay rate (day^−1^)
$\sigma_{4}$	1 [80,81]	Interferon clearance rate (day^−1^)
$f_{0}$	1900 [35]	Virus production
		rate [(cells· day)^−1^ (copies/mL)]
$f_{1}$	0.001	Rate of interferon influence in virus
		production [(pg/mL)^−1^]
κ	0.1	Coefficient of infected ECs that
		stimulates the Interferon secretion
$g_{0}$	500 [35]	Interferon secretion rate [(pg/mL) (cells · day)^−1^]
$g_{1}$	$7.5\cdot 10^{-6}$	Rate of virus influence in interferon
		production [(copies/mL)^−1^]

**Table A2:** Parameters for adaptive immune response.

Parameter	Value	Definition
$h_{0}$	1	Naive T-lymphocytes production
		rate cells/day
$h_{1}^{0}$	1.51 [37,78]	T-helper cells (CD4+) differentiation
		rate [(cells · day)^−1^]
$h_{1}^{1}$	0.001	T-helper cells (CD4+) differentiation
		rate (cells^−1^)
$h_{2}^{0}$	0.85	Cytotoxic T cells (CD8+) differentiation
		rate [(cells · day)^−1^]
$h_{2}^{1}$	0.1	Cytotoxic T cells (CD8+) differentiation
		rate (cells^−1^)
$q_{0}$	1	Naive B-lymphocytes production
		rate cells/day
$q_{1}^{0}$	$6.25\cdot 10^{-3}$	Effector B-cells differentiation
		rate [(cells · day)^−1^]
$q_{1}^{1}$	$1.68\cdot 10^{-2}$	Effector B-cells differentiation
		rate (cells^−1^)
$\sigma_{5}$	0.023 [82]	T-helper cells (CD4+) elimination
		rate (day^−1^)
$\sigma_{6}$	0.031 [82]	Cytotoxic T cells (CD8+) elimination
		rate (day^−1^)
$\sigma_{7}$	0.028 [83]	Effector B-cells elimination
		rate (day^−1^)
$\sigma_{8}$	0.04 [35,37,79]	Antibodies decay
		rate (day^−1^)
$k_{5}$	1205.63	Antibodies secretion
		rate [(cells·day)^−1^ (units/mL)]
$\gamma_{1}$	$1.19\cdot 10^{-3}$ [37,77]	Killing rate of infected epithelial cells
		by $T_{8}$ [(cells · day)^−1^]
$\gamma_{2}$	0.01	Killing rate of infected APCs
		by $T_{8}$ [(cells · day)^−1^]
$\gamma_{3}$	0.004 [37]	Rate constant of virus neutralization by
		unit antivirus antibody
		[(day)^−1^ (copies or units/mL)]

**Table A3:** Parameters for cytokine storm.

Parameter	Value	Definition
$r_{1}$	3	Antigen presenting cells production rate by
		cytokines [(cells/day) (pg/mL)^−1^]
$r_{2}$	0.1	Antigen presenting cells production rate by
		cytokines [(pg/mL)^−1^]
$r_{3}$	1	Pro-inflammatory cytokines
		secretion rate [(cells · day)^−1^]
$r_{4}$	0.1	Pro-inflammatory cytokines
		secretion rate [(pg/mL)^−1^]
$p_{1}$	0.4	Pro-inflammatory cytokines secretion rate by
		virus [(pg) (copies · day)^−1^]
$p_{2}$	10	Rate of virus influence in cytokines
		secretion [(copies/mL)^−1^]
$p_{3}$	0.2	Rate of interferon influence in cytokines
		secretion [(pg/mL)^−1^]
$\sigma_{S}$	0.25 [84]	Pro-inflammatory cytokines elimination rate (day^−1^)

**Table A4:** Variables, definitions and initial values.

Variable	Definition	Initial Condition
*E*	Uninfected epithelial cells (cells/mL)	5 · 10${}^{5}$ [37]
$E_{v}$	Infected epithelial cells (cells/mL)	0
*C*	Uninfected dendritic cells	0 and 10${}^{3}$ [37]
	and macrophages (cells/mL)	
$C_{v}$	Infected dendritic cells	0
	and macrophages (cells/mL)	
*V*	Virus load (copies/mL)	it varies
*I*	Interferon (pg/mL)	0
$T_{n}$	Naive T-lymphocytes cells	2 · 10${}^{3}$
$T_{4}$	T-helper cells	0
$T_{8}$	T-killer cells	0
$B_{n}$	Naive B-lymphocytes cells	1 · 10${}^{3}$ [37]
*B*	Plasma cells	0
*A*	Antiviral antibody titer	0
*S*	Pro-inflammatory cytokines (pg/mL)	0
